# Inhibition of HMGB1 release via salvianolic acid B-mediated SIRT1 up-regulation protects rats against non-alcoholic fatty liver disease

**DOI:** 10.1038/srep16013

**Published:** 2015-11-03

**Authors:** Wenjing Zeng, Wen Shan, Lili Gao, Dongyan Gao, Yan Hu, Guangzhi Wang, Ning Zhang, Zhenlu Li, Xiaofeng Tian, Wei Xu, Jinyong Peng, Xiaochi Ma, Jihong Yao

**Affiliations:** 1 Department of Pharmacology, Dalian Medical University, Dalian, 116044, China; 2 Department of Pharmacy, Second Affiliated Hospital of Dalian Medical University, Dalian, 116023, China; 3 Department of General Surgery, Second Affiliated Hospital of Dalian Medical University, Dalian, 116023, China; 4 Department of General Surgery, Ruijin Hospital, Shanghai Jiaotong University School of Medicine, Shanghai, China

## Abstract

The inflammatory mediator high-mobility group box 1 (HMGB1) plays a critical role in the pathogenesis of non-alcoholic fatty liver disease (NAFLD). However, the regulation of HMGB1 in NAFLD, particularly through sirtuin 1 (SIRT1), remains unclear. In this study, we investigated the role of SIRT1-mediated inhibition of HMGB1 release in NAFLD and the effect of salvianolic acid B (SalB), which is a water-soluble phenolic acid extracted from Radix *Salvia miltiorrhiza*, on NAFLD through SIRT1/HMGB1 signaling. *In vivo*, SalB treatment significantly attenuated high-fat diet (HFD)-induced liver damage, hepatic steatosis, and inflammation. Importantly, SalB significantly inhibited HMGB1 nuclear translocation and release, accompanied by SIRT1 elevation. In HepG2 cells, palmitic acid (PA)-induced pro-inflammatory cytokines release were blocked by HMGB1 small interfering RNA (siRNA) transfection. Moreover, pharmacological SIRT1 inhibition by Ex527 induced HMGB1 translocation and release, whereas SIRT1 activation by resveratrol or SalB reversed this trend. SIRT1 siRNA abrogated the SalB-mediated inhibition of HMGB1 acetylation and release, suggesting that SalB-mediated protection occurs by SIRT1 targeting HMGB1 for deacetylation. We are the first to demonstrate that the SIRT1/HMGB1 pathway is a key therapeutic target for controlling NAFLD inflammation and that SalB confers protection against HFD- and PA-induced hepatic steatosis and inflammation through SIRT1-mediated HMGB1 deacetylation.

Non-alcoholic fatty liver disease (NAFLD) is the leading cause of chronic liver disease and has emerged as a growing public health problem worldwide^1^. NAFLD encompasses a disease spectrum ranging from simple steatosis to non-alcoholic steatohepatitis (NASH), fibrosis, cirrhosis, and hepatocellular carcinoma (HCC)^2^. However, the mechanisms involved in the pathogenesis of NAFLD are not yet fully understood. Consolidated data have suggested that inflammation may play a critical role in the pathogenesis of NAFLD^3^,^4^,^5^. Inflammation, which is caused by a “second hit” combined with a primary insult (the “first hit,” which triggers steatosis), leads to progressive disease. As the specific management that is available for the prevention of NAFLD is limited, efforts to achieve a better understanding of the mechanisms controlling hepatic steatosis and inflammation in NAFLD are crucial to the development of new therapeutic strategies.

High-mobility group box 1 (HMGB1) is a highly conserved nuclear protein that can regulate gene transcription and maintain nucleosome structure^6^. In addition to its nuclear roles, HMGB1 also functions as an inflammatory cytokine when passively released from necrotic cells or actively secreted from stressed cells^7^. As an inflammatory mediator, HMGB1 plays a crucial role in various forms of liver disease, including steatosis, inflammation, fibrosis and tumorigenesis^8^,^9^,^10^. Recent studies have indicated that the HMGB1 level is increased in NAFLD in both animals^11^,^12^,^13^ and the clinic^14^,^15^ and that HMGB1 inhibition results in a significant reduction in the inflammatory response in NAFLD^9^,^16^. These findings suggest that HMGB1 may be an important mediator in NAFLD and that inhibition of HMGB1 may represent a potential approach for anti-inflammatory therapy of NAFLD.

Several post-translational modifications, including phosphorylation, methylation and acetylation, are involved in the relocation of HMGB1 to the cytoplasm and its subsequent secretion^17^,^18^,^19^. The hyperacetylation of HMGB1 affects its DNA binding and redirects it toward the cytoplasm and secretion^19^,^20^. Recent studies have demonstrated that HMGB1 is acetylated and released from hepatocytes in liver ischemia/reperfusion (I/R) and have also identified decreased HDAC1 and HDAC4 as critical for regulating acetylated HMGB1 release from neurons in response to ethanol exposure^21^,^22^. However, it is unknown whether the acetylation of HMGB1 regulates its release from hepatocytes during the pathogenesis of NAFLD.

Mammalian sirtuin 1 (SIRT1), an NAD-dependent class III histone deacetylase (HDAC), plays important roles in many physiological processes, including gene transcription, senescence, energy metabolism, oxidative stress and inflammation^23^,^24^. Previous studies have demonstrated that hepatocyte-specific SIRT1-knockout mice and SIRT1+/– mice are more prone to suffering from liver steatosis and inflammation than wild-type mice^25^,^26^, suggesting that SIRT1 exerts anti-steatosis and anti-inflammatory activities during the pathogenesis of NAFLD. Our most recent research demonstrated that SIRT1 modulates HMGB1 hyperacetylation and extracellular release^27^. Therefore, we hypothesized that SIRT1 may suppress HMGB1 release in high-fat diet (HFD)-induced NAFLD, thereby affording a novel target for anti-inflammatory therapy for NAFLD.

Salvianolic acid B (SalB) is one of the most efficacious water-soluble phenolic acids that are extracted from Radix *Salvia miltiorrhiza* (Danshen). Previous studies have suggested that SalB is an effective agent for reversing liver fibrosis^28^. We recently demonstrated that SalB can attenuate acute ethanol-induced hepatocyte apoptosis and hepatic inflammation via SIRT1 activation^29^. However, the role of SalB in NAFLD is unknown.

The purposes of the present study were to investigate whether SIRT1-mediated HMGB1 deacetylation can modulate the release of HMGB1 during the progression of NAFLD and to explore whether SalB can protect against NAFLD via the SIRT1/HMGB1 pathway.

## Results

### SalB diminishes HFD-induced liver injury and liver steatosis

**Figure 1:**
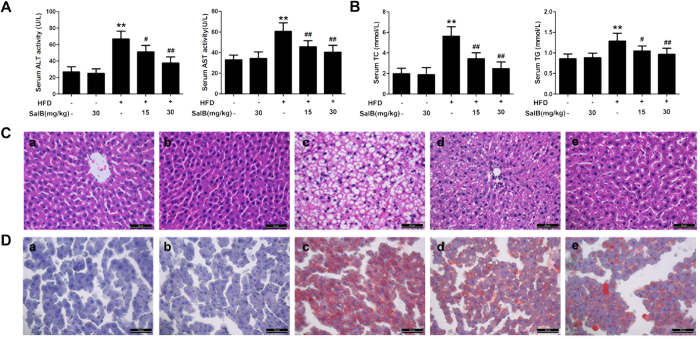
SalB diminishes HFD-induced liver injury and hepatic steatosis. (**A**) Serum levels of alanine aminotransferase (ALT) and aspartate aminotransferase (AST). (**B**) Serum levels of total cholesterol (TC) and triglyceride (TG). The results are the mean ± SD (n = 8), ^**^P < 0.01 vs. the control group, ^#^*P *< 0.05 vs. the HFD group, ^##^*P *< 0.01 vs. the HFD group. (**C**,**D**) H&E staining and Sudan IV staining of liver sections from the experimental groups: a, control; b, control + SalB (30 mg/kg); c, HFD; d, HFD + SalB (15 mg/kg); and e, HFD + SalB (30 mg/kg). H&E- and Sudan IV-stained sections were photographed at 400× magnification.

We first determined whether SalB plays a protective role in HFD-induced NAFLD. As shown in Fig. 1A, serum alanine aminotransferase (ALT) and aspartate aminotransferase (AST) levels in the HFD group were clearly increased compared with those in the control group and the SalB control group. SalB treatment remarkably inhibited ALT and AST activities in a dose-dependent manner (*P* < 0.01) but did not affect ALT and AST levels in the control rats, suggesting a protective effect of SalB against HFD-induced liver injury. In addition, compared with the control group, the serum levels of total cholesterol (TC) and triglyceride (TG), which are sensitive serum biomarkers of liver steatosis, were remarkably increased in the HFD group, while SalB treatment reversed this trend (Fig. 1B). In agreement, both H&E staining and Sudan IV staining of liver sections showed the accumulation of lipid droplets in the livers of the HFD-fed animals, whereas lipid droplets were rare in livers of the control animals and the rats that were treated with SalB alone (Fig. 1C,D). However, when the HFD-fed rats were co-administered SalB, the histology showed less accumulation of hepatic lipid droplets. Collectively, these results suggest that SalB is an effective agent in protecting rats from HFD-induced liver injury and liver steatosis.

### SalB suppresses the release of pro-inflammatory cytokines

**Figure 2:**
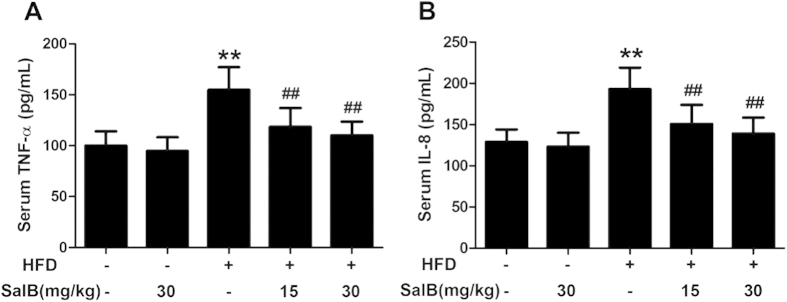
SalB suppresses the release of pro-inflammatory cytokines. (**A**) Serum tumor necrosis factor-α (TNF-α). (**B**) Serum interleukin-8 (IL-8). The results are the mean ± SD (n = 8), ^**^P < 0.01 vs. the control group, ^##^*P *< 0.01 vs. the HFD group.

Serum TNF-α and IL-8 levels are elevated in NAFLD^30^,^31^. Indeed, we found that the serum TNF-α and IL-8 levels were remarkably increased in the HFD group, whereas SalB treatment prevented the increase in these inflammatory cytokine levels (Fig. 2A,B).

### SalB mediates protection against HFD- and palmitic acid (PA)-induced hepatic steatosis and inflammation through SIRT1 up-regulation

**Figure 3:**
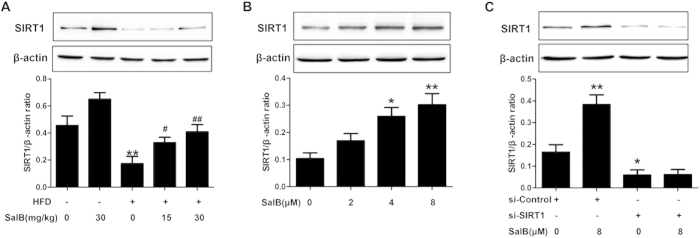
Effects of SalB on the SIRT1 protein level *in vivo* and *in vitro*. (**A**) Western blot analysis of the hepatic SIRT1 protein level in rats. ^**^P < 0.01 vs. the control group, ^#^*P *< 0.05 vs. the HFD group, ^##^*P *< 0.01 vs. the HFD group (n = 3). (**B**) HepG2 cells were pretreated with 2, 4 or 8 μM SalB for 3 h. The figure shows a dose-dependent response of SIRT1 to SalB treatment. ^*^P < 0.05 vs. the control group, ^**^P < 0.01 vs. the control group. (**C**) HepG2 cells were transfected with control siRNA or SIRT1 siRNA, and the transfected cells were exposed to 8 μM SalB for 3 h. The SIRT1 protein level was then measured by Western blotting. ^*^P < 0.05 vs. the si-Control group, ^**^P < 0.01 vs. the si-Control group (n = 3).

Hepatic SIRT1 expression is reduced in different animal models of NAFLD, and loss of SIRT1 in hepatocytes leads to the development of hepatic steatosis and inflammation while on an HFD^32^,^33^, suggesting that pharmacological activation of SIRT1 may constitute a potential therapeutic strategy. Based on our previous study, we hypothesized that SalB exerted its “anti-steatosis” and “anti-inflammatory” effects by up-regulation of SIRT1. As shown in Fig. 3A, the hepatic SIRT1 protein level was remarkably reduced in HFD-fed rats, and SalB reversed this loss of SIRT1. Given that previous observations have documented that liver parenchymal cells are imperative in the early stage of NAFLD^9^, we used HepG2 cells to further confirm that SalB treatment causes SIRT1 up-regulation *in vitro*. SalB triggered a dose-dependent increase in SIRT1 expression, whereas SalB-mediated SIRT1 up-regulation was mostly abrogated upon SIRT1 small interfering RNA (siRNA) transfection in HepG2 cells (Fig. 3B,C).

**Figure 4:**
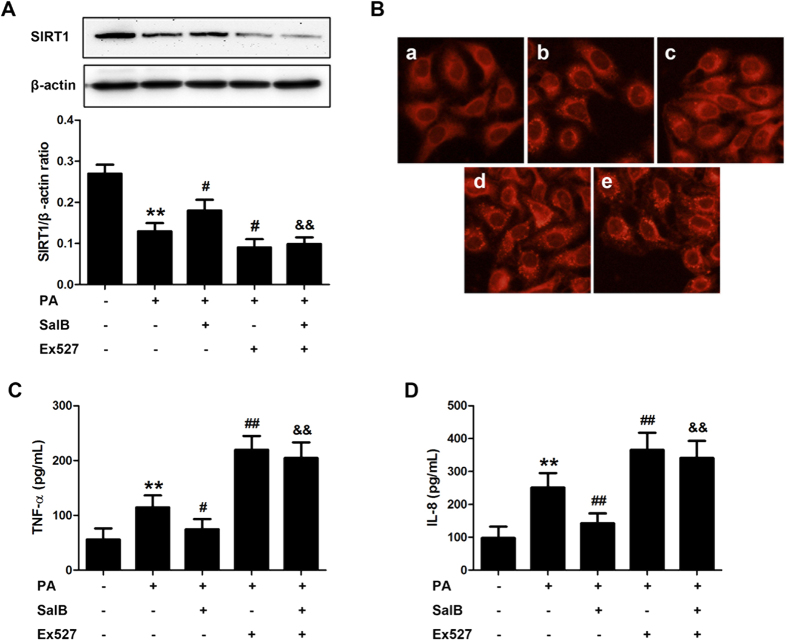
SIRT1 activation attenuates hepatic steatosis and the release of pro-inflammatory cytokines induced by PA in HepG2 cells. HepG2 cells were pretreated with 8 μM SalB for 3 h and/or 10 μM Ex527 for 6 h before being exposed to PA. (**A**) The protein level of SIRT1 was evaluated by Western blotting. ^**^P < 0.01 vs. the control group, ^#^*P *< 0.05 vs. the PA group, ^&&^*P *< 0.01 vs. the PA group pretreated with 8 μM SalB (n = 3). (**B**) Nile Red staining (200×): **a**, control group; **b**, PA group (500 μM PA for 24 h); **c**, PA group pretreated with 8 μM SalB; **d**, PA group pretreated with 10 μM Ex527;**e**, PA group pretreated with 8 μM SalB and 10 μM Ex527. (**C**) The TNF-α and (**D**) IL-8 levels in the culture medium were measured by ELISA. The results are the mean ± SD (n = 6), ^**^P < 0.01 vs. the control group, ^#^*P *< 0.05 vs. the PA group, ^##^*P *< 0.01 vs. the PA group, ^&&^*P* < 0.01 vs. the PA group pretreated with 8 μM SalB.

To mimic NAFLD *in vivo*, a PA-induced cell model was established as previously described^34^. To evaluate whether SIRT1 contributed to SalB-mediated protection *in vitro*, HepG2 cells were pretreated with SalB and/or the specific SIRT1 antagonist Ex527^35^ before exposure to PA. As shown in Fig. 4, HepG2 cells that were treated with PA for 24 h exhibited a decrease in SIRT1 (Fig. 4A), an increase in lipid droplets (Fig. 4B) and an increase in pro-inflammatory cytokines (Fig. 4C,D). SalB treatment significantly up-regulated SIRT1 and consequentially reversed the accumulation of lipid droplets and the secretion of TNF-α and IL-8, whereas the SalB-mediated protection was significantly blocked by Ex527. In addition, the levels of HMGB1, IL-1β, TNF-α and IL-8 in the culture medium from primary Kupffer cells (KCs) was remarkably increased in the PA group, and SalB treatment prevented the increase in the levels of these inflammatory cytokines (Supplementary Fig. S1). Thus, these results suggest that SalB-induced protection against HFD- and PA-induced hepatic steatosis and inflammation is mediated by SIRT1 up-regulation.

### SalB inhibits HMGB1 nuclear translocation and release through up-regulation of SIRT1

**Figure 5:**
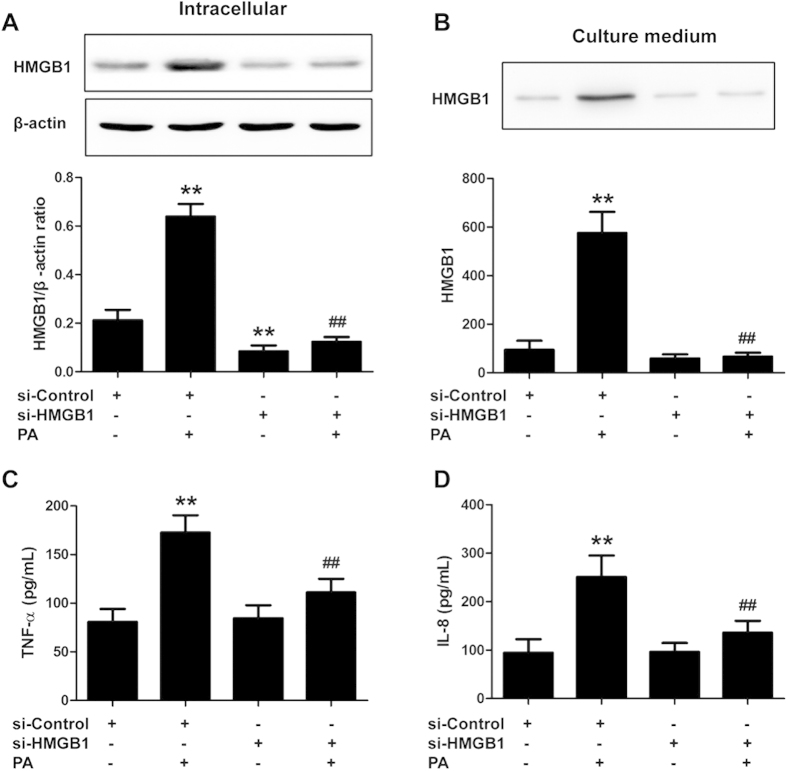
HMGB1 inhibition attenuates the release of pro-inflammatory cytokines induced by PA in HepG2 cells. HepG2 cells were transfected with a control siRNA or HMGB1 siRNA before being exposed to PA. The HMGB1 proteins in (**A**) the whole-cell lysate and (**B**) the culture medium were measured by Western blotting. ^**^P < 0.01 vs. the si-Control group, ^##^*P *< 0.01 vs. the PA group pretreated with control siRNA (n = 3). (**C)** TNF-α and (**D**) IL-8 levels in the culture medium were measured by ELISA. The results are the mean ± SD (n = 6), ^**^P < 0.01 vs. the si-Control group, ^##^*P *< 0.01 vs. the PA group pretreated with control siRNA.

HMGB1, a central and necessary inflammatory mediator, has been shown to be highly increased in HFD-induced NAFLD. To ascertain the role of HMGB1 in NAFLD in the current study, we knocked down HMGB1 in HepG2 cells using siRNA before exposure to PA. As shown in Fig. 5 and Supplementary Fig. S2, HepG2 cells that were treated with PA for 24 h exhibited an increase in the expression of HMGB1 and the release of HMGB1, IL-1β, TNF-α and IL-8. While PA-induced HMGB1 expression and pro-inflammatory cytokines release were blocked by HMGB1 siRNA. These results demonstrate that HMGB1 plays a critical role in the development of PA-induced NAFLD inflammation.

**Figure 6:**
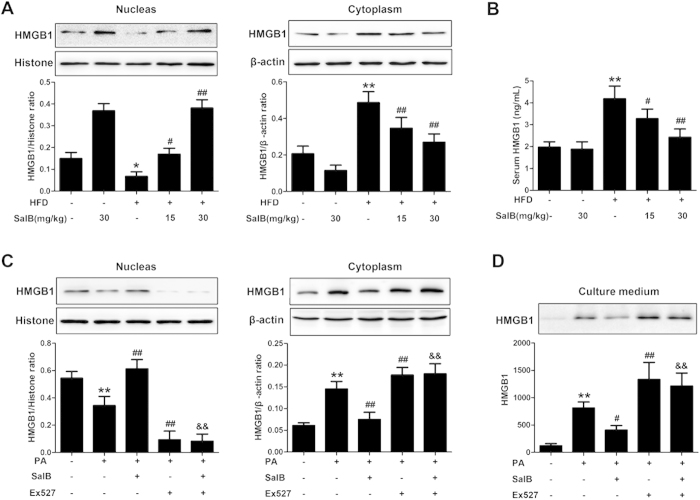
SalB inhibits HMGB1 nuclear translocation and release through up-regulation of SIRT1. (**A**) The levels of nuclear and cytoplasmic HMGB1 in rat livers were measured by Western blotting. ^*^P < 0.05 vs. the control group, ^**^P < 0.01 vs. the control group, ^#^*P *< 0.05 vs. the HFD group, ^##^*P *< 0.01 vs. the HFD group (n = 3). (**B**) The serum levels of HMGB1 in the rats were measured by ELISA. The results are the mean ± SD (n = 8), ^**^P < 0.01 vs. the control group, ^#^*P *< 0.05 vs. the HFD group, ^##^*P *< 0.01 vs. the HFD group. (**C**,**D**) HepG2 cells were pretreated with SalB for 3 h or with Ex527 for 6 h and then subjected to 500 μM PA for 24 h. The levels of HMGB1 in the nucleus, cytoplasm and culture medium were then evaluated by Western blotting. ^**^P < 0.01 vs. the control group, ^#^*P *< 0.05 vs. the PA group, ^##^*P *< 0.01 vs. the PA group, ^&&^*P *< 0.01 vs. PA group pretreated with 8 μM SalB (n = 3).

Next, we further explored the effect of SalB on the translocation and release of HMGB1 in NAFLD. The translocation of HMGB1 from the nucleus to the cytoplasm was observed in the livers of HFD-fed rats compared with the control group, whereas SalB reversed this trend (Fig. 6A). In addition, the serum HMGB1 level was markedly up-regulated in the HFD-fed group, and notably, SalB significantly reduced the release of HMGB1 from hepatocytes (Fig. 6B). In accordance with *in vivo* data, the translocation of HMGB1 from the nucleus to the cytoplasm in HepG2 cells and the release of HMGB1 into the supernatants of HepG2 cells were dramatically elevated after 24 h of PA treatment. SalB significantly inhibited this translocation and release of HMGB1, while SalB-mediated inhibition was significantly blocked by Ex527 (Fig. 6C,D). Taken together, our findings indicate that SalB inhibits the nuclear translocation and release of HMGB1 via up-regulation of SIRT1.

### SalB-mediated protection depends on SIRT1 targeting HMGB1 for deacetylation

**Figure 7:**
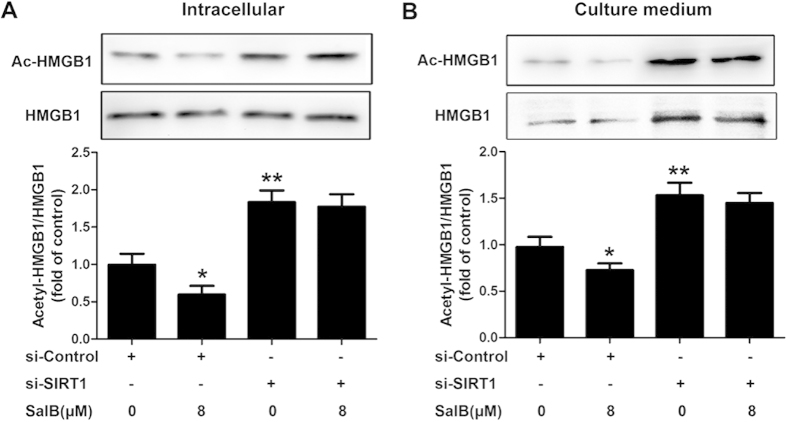
SalB-mediated protection is dependent on SIRT1 targeting HMGB1 for deacetylation. HepG2 cells were transfected with a control siRNA or SIRT1 siRNA and were subsequently exposed to 8 μM SalB for 3 h. The HMGB1 proteins in the whole-cell lysate and the culture medium were immunoprecipitated with an anti-HMGB1 antibody and immunoblotted with an anti-acetyl antibody. ^*^P < 0.05 vs. the si-Control group, ^**^P < 0.01 vs. the si-Control group (n = 3).

Previous findings demonstrated that the hyperacetylation of HMGB1 affects its translocation and extracellular secretion^19^,^20^. We thus examined whether the process of HMGB1 translocation and release is regulated by SIRT1-mediated deacetylation. In particular, to assess whether SalB-induced protection is mediated by SIRT1 through targeting HMGB1 for deacetylation, we examined the effect of SalB on the status of HMGB1 acetylation following SIRT1 siRNA treatment of HepG2 cells. As shown in Fig. 7A, the knockdown of SIRT1 increased the acetylation of HMGB1 compared to that of control siRNA, while SalB reduced the levels of acetylated HMGB1 in the cells, and SalB-mediated down-regulation of acetylated HMGB1 was abolished by SIRT1 siRNA. In contrast to the control siRNA treatment, SIRT1 knockdown markedly elevated the release of HMGB1 and acetylated HMGB1 into the culture medium, and there was an obvious change in the proportion of acetylated HMGB1. However, SalB counteracted the release of HMGB1 and significantly reduced the proportion of acetylated HMGB1 in the culture medium, and the SalB-mediated down-regulation of acetylated HMGB1 was blocked by SIRT1 siRNA (Fig. 7B). These data demonstrate that the SalB-mediated inhibition of HMGB1 acetylation and release is partly achieved through up-regulation of SIRT1.

### SalB suppresses hepatic inflammation through the SIRT1/HMGB1 pathway

**Figure 8:**
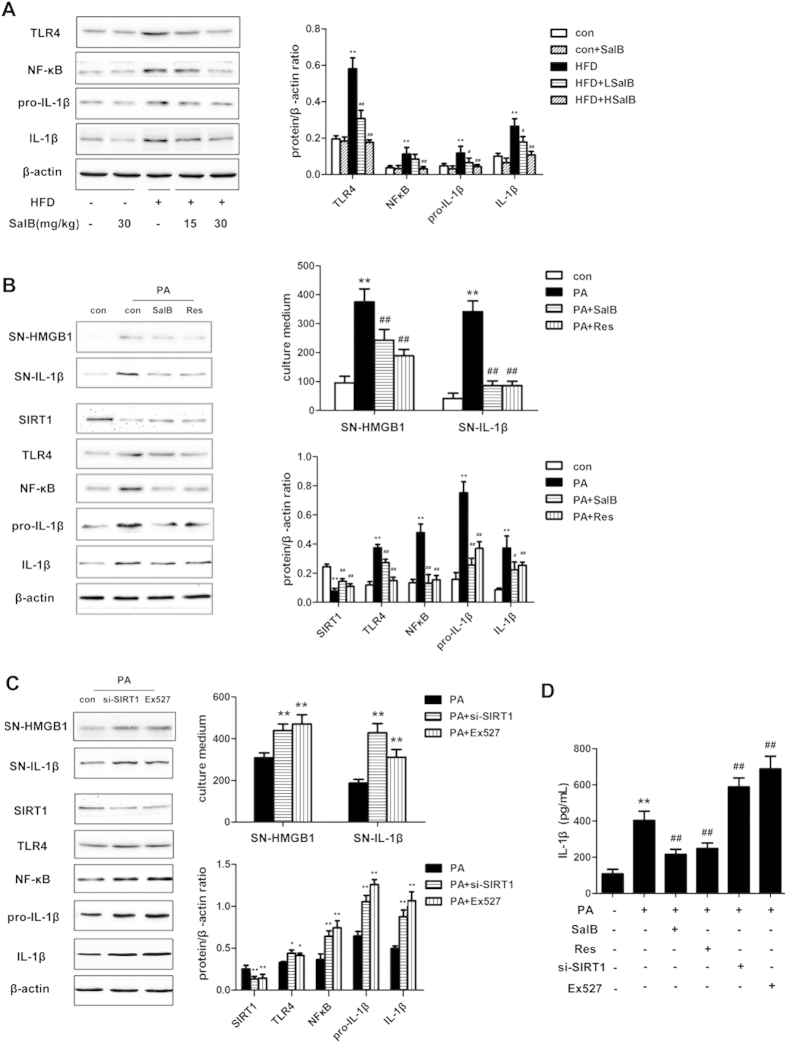
Effects of SalB on TLR4, NF-κB and IL-1β expression *in vivo* and *in vitro*. (**A**) The protein levels of SIRT1, TLR4, NF-κB, pro-IL-1β and IL-1β in the rat livers were measured by Western blotting. ^**^P < 0.01 vs. the control group, ^#^*P *< 0.05 vs. the HFD group, ^##^*P *< 0.01 vs. the HFD group (n = 3). (**B**) HepG2 cells were pretreated with 8 μM SalB or 10 μM resveratrol (Res) and then exposed to PA for 24 h. The expression of SIRT1, TLR4, NF-κB, pro-IL-1β and IL-1β proteins in HepG2 cells and HMGB1 and IL-1β in the cell culture medium was measured by Western blotting (n = 3). ^**^P < 0.01 vs. the control group, ^#^*P *< 0.05 vs. the PA group, ^##^*P *< 0.01 vs. the PA group. (**C**) HepG2 cells were transfected with SIRT1 siRNA or 10 μM Ex527 and then exposed to PA. The expression of SIRT1, TLR4, NF-κB, pro-IL-1β and IL-1β proteins in HepG2 cells and HMGB1 and IL-1β in the cell culture medium was measured by Western blotting (n = 3). ^*^P < 0.05 vs. the PA group, ^**^P < 0.01 vs. the PA group. (**D**) HepG2 cells were pretreated with 8 μM SalB, 10 μM resveratrol (Res), SIRT1 siRNA or 10 μM Ex527 and then exposed to PA for 24 h. The level of IL-1β in the culture medium was measured by ELISA. ^**^P < 0.01 vs. the control group, ^##^*P *< 0.01 vs. the PA group (n = 6).

It has been suggested that inflammation-related factors, such as Toll-like receptor-4 (TLR4), nuclear factor-κB (NF-κB), and IL-1β, play important roles in the progression of HFD-induced NAFLD^9^,^36^,^37^. Therefore, we investigated changes in these proteins to determine whether SalB treatment alleviated the inflammation in the HFD-fed rats. As shown in Fig. 8A, the HFD-induced increase of liver TLR4, NF-κB, pro-IL-1β and IL-1β proteins was inhibited by SalB treatment. We further investigated the molecular mechanism by which SalB protects hepatocytes from PA-induced hepatic inflammation *in vitro*. HepG2 cells treated with PA exhibited decreased the expression of SIRT1 but increased the release of HMGB1 and IL-1β. In contrast, both SalB and resveratrol^38^, a well-known SIRT1 activator, increased the expression of SIRT1 and decreased the release of HMGB1 and IL-1β compared with PA treatment (Fig. 8B,D). Furthermore, pretreatment with SalB or resveratrol reduced the PA-induced up-regulation of TLR4, NF-κB, pro-IL-1β and IL-1β. Similar to the effect of SalB and resveratrol, HMGB1 siRNA decreased the PA-induced release of HMGB1 and related pro-inflammatory cytokines (Supplementary Fig. S2). The above data indicate that SalB suppresses hepatic inflammation through the SIRT1/HMGB1 pathway.

Next, to ascertain the role of SIRT1 in HMGB1-triggered inflammation, an siRNA strategy was applied. Both siRNA- and Ex527-mediated SIRT1 knockdown increased the release of HMGB1 and IL-1β. In agreement with this result, SIRT1 knockdown also increased the expression of TLR4, NF-κB, pro-IL-1β and IL-1β (Fig. 8C,D). These results indicate that the HMGB1 release induced by inhibition of SIRT1 dramatically aggravates PA-induced hepatic inflammation.

## Discussion

As currently available therapeutic approaches to NAFLD show rather limited effectiveness, novel treatment strategies are required. The present study represents the first attempt to demonstrate that 1) the SIRT1/HMGB1 pathway is a pivotal therapeutic target for preventing NAFLD progression, 2) SalB confers protection against HFD- and PA-induced hepatic steatosis and inflammation, and 3) the protective effect of SalB may be associated with the SIRT1/HMGB1 pathway.

*Salvia miltiorrhiza*, also known as Danshen, has been widely used for thousands of years in traditional Chinese medicine, with little reported toxicity. SalB, the most abundant and bioactive water-soluble compound that is isolated from *Salvia miltiorrhiza*, possesses a variety of pharmacological effects, such as improvement of vascular function^39^, reduction of obesity-associated metabolic disorders^40^, attenuation of hepatocyte apoptosis^41^ and reversal of liver fibrosis^28^,^36^. Furthermore, we recently reported that SalB protects against acute ethanol-induced liver injury^29^. To our knowledge, the present study is the first to report that SalB exerts potent hepatoprotective functions in a dietary rat model of NAFLD. We found that SalB reduced the serum ALT and AST levels in rats with HFD-induced NAFLD. Improvement in the HFD-induced elevation of serum TG and TC was accompanied by a considerable reduction in hepatic steatosis. SalB treatment further demonstrated the ability to down-regulate essential inflammatory factors. Thus, SalB has highly favorable characteristics for the treatment of NAFLD.

A growing number of studies have demonstrated that SIRT1 plays a central role in the pathogenesis of NAFLD. However, an HFD decreases SIRT1 expression, and hepatocyte-specific deletion of SIRT1 leads to hepatic steatosis and inflammation^25^,^26^. Several pharmacological SIRT1 activators have thus been developed for the treatment of NAFLD^32^,^42^. Importantly, we have previously demonstrated that SalB is a potent activator of SIRT1^29^. In this study, SIRT1 expression in the liver significantly decreased in the NAFLD model. However, SalB enhanced the expression of SIRT1, suggesting that SalB may confer protection against HFD-induced NAFLD through SIRT1 up-regulation. To evaluate such a possibility, the SIRT1 siRNA strategy was used in HepG2 cells. As expected, the results indicated that the knockdown of SIRT1 enhanced the PA-induced reduction in SIRT1. Moreover, when a specific inhibitor of SIRT1 (Ex527) was administered to PA-treated HepG2 cells, the SalB-induced positive regulatory effect on SIRT1 was significantly inhibited. Thus, SIRT1 up-regulation by SalB prevents HFD- and PA-induced NAFLD.

Because hepatocyte inflammation plays a vital role in the pathogenesis of many chronic liver diseases, we further explored the anti-inflammatory pathway, which may be related to SIRT1. HMGB1 has been a top SIRT1 substrate candidate in peptide microarray experiments^43^. This nuclear protein participates in chromatin architectural remodeling and transcriptional regulation. When secreted from cells, HMGB1 can also act as a pro-inflammatory mediator or alarmin. In most cases, the cells that actively secrete HMGB1 appear to be immune cells, such as macrophages, natural killer cells, and dendritic cells^19^,^44^. However, it is becoming increasingly clear that nonimmune parenchymal cells also participate in active HMGB1 secretion. In the early stage of NAFLD in particular, the main source of secreted HMGB1 is liver parenchymal cells^9^. Extracellular HMGB1 is responsible for the inflammatory response to hepatic injury, as shown in models of liver I/R and alcoholic liver disease^22^,^45^,^46^. Recent studies also indicated that the level of HMGB1 is increased in experimental models of NAFLD^11^,^12^ and in patients with NAFLD^13^,^14^,^15^. HMGB1 serves as an early mediator, integrating and enhancing the TLR4/MyD88-dependent pathway to accelerate HFD-induced liver damage and inflammation during the early stage of NAFLD^9^. As a result, anti-HMGB1 treatment can significantly reduce the production of cytokines upon PA or HFD treatment, resulting in inhibition of hepatic inflammation^9^,^16^. In our study, siRNA-mediated HMGB1 knockdown markedly reduced the PA-induced increase in the expression of HMGB1 and the release of HMGB1, TNF-α, IL-8 and IL-1β, confirming the crucial role played by HMGB1 in PA-induced NAFLD inflammation. Moreover, the translocation and release of HMGB1 markedly increased in the HFD group, whereas pretreatment with SalB decreased the translocation and release of HMGB1 in a dose-dependent manner. In parallel, SalB significantly inhibited PA-induced HMGB1 release in HepG2 cells. However, Ex527 increased the release of HMGB1. Collectively, these results indicate that SalB can inhibit the relocation and release of HMGB1 through up-regulation of SIRT1 in liver parenchymal cells during NAFLD.

To study the protective role of SalB in HMGB1-mediated inflammation, we also evaluated the expression of TLR4, NFκB and IL-1β. As a functional receptor of HMGB1, TLR4 can promote the inflammatory processes through NF-κB activation and exhibits an increase in protein expression in NAFLD^8^,^9^,^12^. In line with previous studies, we found that PA or HFD increased the expression of TLR4 and NF-κB, whereas SalB pretreatment reversed this trend. Apart from TLR4, HMGB1 also transduces cellular signal through the receptor for advanced glycation end products (RAGE), which plays an important role in the development of diet-induced NAFLD and obesity in different manners^47^. Further study is needed to explore whether SalB has an effect on this HMGB1 receptor. In addition to being a receptor of HMGB1, TLR4 is an important receptor of LPS. Serum LPS level was significantly elevated, which might trigger liver injury through binding to TLR4 in NASH^48^,^49^. Recent evidence suggested that overgrowth of intestinal bacteria and increased intestinal permeability are associated with the development of NASH; however, causality has not been proven and mechanistic links require further delineation^50^. Hence, further investigation is still warranted to explore whether inflammation resulted from HMGB1 alone or not. Previous reports have also indicated that IL-1β can form complexes with HMGB1 to enhance the stimulatory capacity^51^, which is of interest regarding NASH pathogenesis, given that IL-1β is an abundant pro-inflammatory cytokine in various diet-induced NASH models^37^. Pro-IL-1β is synthesized as a 31 kD proform that can be cleaved into the mature 17 kD secreted form. Consistently, accompanied by inhibition of HMGB release, pretreatment with SalB significantly reduced the expression of TLR4, NF-κB, pro-IL-1β and IL-1β and the release of IL-1β as induced by HFD. Therefore, it can be concluded that HMGB1 plays a vital role in hepatocyte inflammation, as induced by HFD or PA exposure.

The acetylation, phosphorylation, and redox control of HMGB1 influence its subcellular location in response to various stimuli. It appears that deacetylation in particular plays a vital role in the regulation of HMGB1 release^19^,^20^,^21^. For instance, a recent study by Evankovich *et al.* revealed that markedly reduced nuclear HDAC1 and HDAC4 activities in hepatocytes following liver I/R promote the hyperacetylation and subsequent release of HMGB1^22^. In addition, PARP-1 regulates the translocation of HMGB1 to the cytoplasm by up-regulating the acetylation of HMGB1 in macrophages^52^. More recently, we observed that the resveratrol-mediated inhibition of HMGB1 nucleo-cytoplasmic translation in sepsis-induced liver injury depends on SIRT1-mediated deacetylation^27^. Similar to our findings, studies by Rabadi *et al.* have demonstrated that the inflammation-induced repression of SIRT1 disables the deacetylation of HMGB1 and facilitates its nuclear-to-cytoplasmic translocation and systemic release, thus maintaining inflammation^53^. Consistent with these observations, we found that SIRT1 siRNA significantly increased HMGB1 hyperacetylation compared with control siRNA, suggesting that SIRT1 is responsible for the inhibition of HMGB1 hyperacetylation. Additionally, SalB-induced SIRT1 up-regulation decreased HMGB1 acetylation in HepG2 cells that were transfected with control siRNA, whereas transfection with SIRT1 siRNA blocked the effect of SalB on acetyl-HMGB1 expression. Together, these data indicate that the SalB-mediated inhibition of HMGB1 nuclear translocation and hyperacetylation is at least partly achieved through up-regulation of SIRT1. The redox modification of cysteine residues determines the pro-inflammatory activity of HMGB1^54^, and the generation of lipid peroxidation and ROS which are increased in the pathogenesis of NAFLD^1^, may partially oxidate HMGB1 and enhance its pro-inflammatory activity. Thus, more experiments should be performed to determine whether SalB, with its anti-oxidation activity, can confer protection against PA/HFD-induced inflammation through affecting the redox status of HMGB1.

In summary, the present study is the first to reveal that SalB has a protective effect against HFD/PA-induced NAFLD. The protective effect of SalB is specifically associated with increased expression of SIRT1, which is accompanied by reduced translocation and release of HMGB1, resulting in a profound reduction in inflammation. These results demonstrate that SalB confers protection against HFD/PA-induced hepatic steatosis and inflammation, at least partly through SIRT1-mediated deacetylation of HMGB1. The anti-inflammatory SIRT1/HMGB1 pathway may thus represent an attractive pharmacological target for the development of new drugs to arrest NAFLD progression. Similar to other polyphenols^55^,^56^, the mechanism by which SalB up-regulates SIRT1 may be associated with AMP-activated protein kinase (AMPK), cAMP signaling, or other factors, so more experiments are needed to explore the precise regulatory mechanism of SalB-mediated SIRT1 activation. Further studies may also elucidate the long-term effects of SalB on more advanced hepatic changes that occur in NAFLD, such as fibrosis, cirrhosis and the development of hepatocellular carcinoma.

## Methods

### Reagents

SalB (purity > 98%) was purchased from Shanghai Winherb Medical Science Co., Ltd. (Shanghai, China). PA and free fatty acid (FFA)-free BSA were obtained from Sigma-Aldrich (St. Louis, MO, USA).

### Animals and treatments

Male Sprague-Dawley rats (200 ± 20 g) were purchased from the Animal Center of Dalian Medical University (Dalian, China). After acclimatization for 1 week, 40 rats were randomly divided into 5 groups of 8 animals each, as follows: 1) control group, 2) control + SalB (30 mg/kg/d) group, 3) HFD group, 4) HFD + SalB (15 mg/kg/d) group, and 5) HFD + SalB (30 mg/kg/d) group. The rats were housed in individual microisolator cages with free access to sterile water and an irradiated standard diet or an HFD^9^ (2% cholesterol, 7% lard, 8.3% yolk, 16.7% sucrose, and 66% standard diet) in a special pathogen-free facility. This HFD diet contains 4.66 kcal/g with an energy composition of 31.59% from fat, 51.73% from carbohydrate, and 16.68% from protein. The rats then underwent intragastric administration of SalB (15 or 30 mg/kg/d) or the same volume of normal saline through a feeding tube for 8 weeks. After exposure to the HFD for 8 weeks, all of the animals were anesthetized using sevoflurane, and then euthanized by exsanguination via the abdominal aorta. Liver tissues and blood samples were collected for analysis. All of the procedures were conducted according to the guidelines of the Institutional Animal Care and Use Committee of Dalian Medical University and were approved by the Institutional Ethics Committee of Dalian Medical University.

### Biochemical assays

Serum samples were separated by centrifugation at 3000 rpm for 10 min and were kept at −20 °C until analysis. The serum levels of ALT, AST, TG and TC were determined with commercial kits from Nanjing Jiancheng Bioengineering Institute (Nanjing, China). All of the procedures were carried out according to the manufacturers’ instructions.

### Liver histology

The isolated left lateral segment of the liver lobes was fixed in 4% paraformaldehyde. Paraffin-embedded liver sections were then stained with hematoxylin-eosin for pathological evaluation. For the detection of neutral lipid accumulation, liver cryosections were stained using Sudan IV.

### Determination of cytokine levels by ELISA

The levels of TNF-α, IL-8 and HMGB1 in the serum and the levels of TNF-α, IL-8, IL-1β and HMGB1 in the culture medium were measured using commercially available ELISA kits from Cusabio Biotech Co., Ltd. (Wuhan, China) according to the manufacturer’s instructions.

### Cell culture and treatment

Human hepatocellular carcinoma cell line HepG2 was cultured in modified Eagle’s medium (MEM) supplemented with 10% (v/v) fetal bovine serum (FBS) (Gibco, CA, USA). The cells were kept at 37 °C in a humidified incubator with 5% CO_2_.

The *in vitro* model of cellular fat accumulation was established by treating HepG2 cells with PA, as previously described^35^. FFA solutions were also prepared as previously described^57^. Briefly, PA (Sigma no. P-0500) and FFA-free BSA (Sigma no. A-6003) were dissolved in NaOH and double-distilled H_2_O, respectively, and then filtered. A 5 mM FFA/5% BSA solution was prepared by complexing an appropriate amount of FFA to 5% BSA in a 60 °C water bath. This solution was then cooled to room temperature and diluted 1:5 in MEM without FBS, with a final concentration of 1 mM FFA/1% BSA. *In vitro*, HepG2 cells were stimulated with 0.5 mM FFA/1% BSA for 24 h. After incubation with 8 μM SalB for 3 h or with 10 mM Ex527 for 6 h, the HepG2 cells were subjected to 0.5 mM FFA/1% BSA stimulation as needed.

### Nile Red staining

Nile Red is a selective fluorescent stain for intracellular lipid droplets. Cells were fixed in 4% paraformaldehyde and stained with Nile Red solution (1 μg/mL) in the dark for 10 min at 37 °C. Lipid-bound Nile Red was observed with a fluorescence microscope.

### Isolation of cytoplasmic and nuclear proteins

Cytoplasmic and nuclear protein fractions from liver tissue or cultured HepG2 cells were prepared using a commercial protein isolation kit (KeyGEN Biotech, Nanjing, China) according to the manufacturer’s instructions.

### Western blot analysis

Equal amounts of proteins were separated by 10–15% SDS-PAGE and transferred to PVDF membranes (Millipore, Bedford, MA, USA). After blocking, the membranes were immunoblotted with primary antibodies that were specific for SIRT1, HMGB1, acetyl-lysine (Abcam Ltd., Cambridge, UK); NF-κB and pro-IL-1β (Proteintech Group, Wuhan, China), IL-1β (Wanlei Biotech, Shenyang, China), and TLR4 (Santa Cruz Biotechnology, Santa Cruz, CA, USA). After washing, the membranes were incubated with the appropriate secondary antibodies. The membranes were then exposed to enhanced chemiluminescence-plus reagents (Beyotime Institute of Biotechnology, Hangzhou, China). The emitted light was captured by a BioSpectrum 410 multispectral imaging system with a Chemi 410 HR camera and analyzed with Gel-Pro Analyzer Version 4.0 (Media Cybernetics, MD, USA).

### Analysis of acetylated HMGB1 by immunoprecipitation

A sufficient amount of anti-HMGB1 antibody (Abcam Ltd., Cambridge, UK) was added to 200 μg protein and gently rotated at 4 °C overnight. Next, the immunocomplex was captured by adding 25 μL protein A + G agarose beads (Beyotime, Shanghai, China) and gently rotating the mixture at 4 °C for 3 h. The mixture was then centrifuged at 1500 × g for 5 min at 4 °C. The precipitate was washed three times with ice-cold phosphate-buffered saline, resuspended in 1× sample buffer and boiled for 5 min to dissociate the immunocomplex from the beads. Finally, the supernatant was collected by centrifugation and subjected to Western blotting.

### siRNA transfection

HepG2 cells were seeded on 6-well plates at a density of 1 × 10^5^ cells/dish. When the confluence reached 50–60%, the HepG2 cells were transfected with a specific SIRT1 siRNA or HMGB1 siRNA (100 nM) or with non-binding control siRNA (100 nM) using Lipofectamine 2000 (Invitrogen, Karlsruhe, Germany) according to the manufacturer’s instructions. The SIRT1 siRNA sequences were sense 5′-CCCUGUAAAGCUUUCAGAAdtdt-3′ and antisense 5′-UUCUGAAAGCUUUACAGGGdtdt-3′ (Genepharma, Shanghai, China). The HMGB1 siRNA sequences were sense 5′-CAGGAGGAAUACUGAACAUdtdt-3′ and antisense 5′-AUGUUCAGUAUUCCUCCUGdtdt-3′.

### Statistical analysis

ll of the statistical analyses were carried out using SPSS 19.0 software. The data are expressed as the mean ± SD. Statistically significant differences among the groups were analyzed using Student’s t-test or one-way ANOVA. *P* values < 0.05 were considered statistically significant.

## Additional Information

**How to cite this article**: Zeng, W. *et al.* Inhibition of HMGB1 release via salvianolic acid B-mediated SIRT1 up-regulation protects rats against non-alcoholic fatty liver disease. *Sci. Rep.* **5**, 16013; doi: 10.1038/srep16013 (2015).

## Supplementary Material

Supplementary Information
