# Supplementary material for: Inhibition of HMGB1 release via salvianolic acid B-mediated SIRT1 up-regulation protects rats against non-alcoholic fatty liver disease
Source: Sci Rep. 2015 Nov 3;5:16013. doi: 10.1038/srep16013 (PMC4630617; doi:10.1038/srep16013)
Supplement: Supplementary Information [file srep16013-s1.pdf]

Supplementary information

Inhibition of HMGB1 release via salvianolic acid B-mediated SIRT1 upregulation protects rats against non-alcoholic fatty liver disease

Wenjing Zeng[1], Wen Shan[1], Lili Gao[1], Dongyan Gao[1], Yan Hu[2], Guangzhi Wang[3], Ning Zhang[2], Zhenlu Li[3], Xiaofeng Tian[3], Wei Xu[4], Jinyong Peng[1], Xiaochi Ma[1], Jihong Yao[1*]

[1]*Department of Pharmacology, Dalian Medical University, Dalian, 116044, China*

[2]*Department of Pharmacy, Second Affiliated Hospital of Dalian Medical University, Dalian, 116023, China*

[3]*Department of General Surgery, Second Affiliated Hospital of Dalian Medical University, Dalian, 116023, China*

[4]*Department of General Surgery, Ruijin Hospital, Shanghai Jiaotong University School of Medicine, Shanghai, China*

*Corresponding author

Dr. Jihong Yao

Department of Pharmacology

Dalian Medical University

Dalian, China

Email: yaojihong65@hotmail.com

Supplementary materials and methods

Primary Kupffer cells isolation. Rat Kuffer cells (KCs) were isolated and cultured as described previously[1]. Non-parenchymal cell (NPC) suspensions were acquired by collagenase in situ collagenase perfusion of the liver and then KCs were isolated by sedimentation by a two-step Percoll gradient centrifugation.

1. Liu, H., Cao, H. & Wu, Z.Y. Isolation of Kupffer cells and their suppressive effects on T lymphocyte growth in rat orthotopic liver transplantation. *World journal of gastroenterology : WJG* **13**, 3133-3136 (2007).

Supplementary Figure 1.

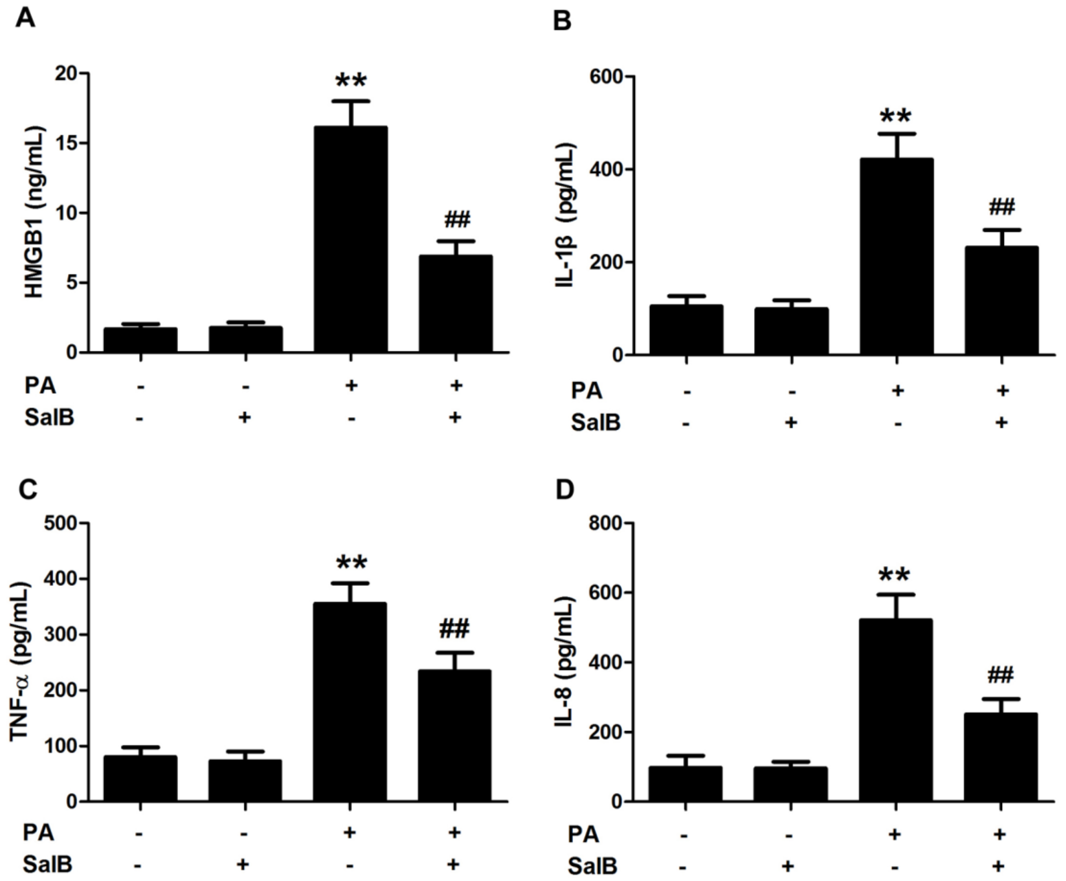

Supplementary figure 1. SalB attenuates the release of HMGB1 and pro-inflammatory cytokines induced by PA in Kupffer cells. Rat Kuffer cells (KCs) were isolated and cultured as described previously [1]. KCs were pretreated with 8 μM SalB for 3 h before being exposed to PA for 24 h. The levels of (A) HMGB1, (B) interleukin-1β (IL-1β), (C) tumor necrosis factor-α (TNF-α), and (D) interleukin-8 (IL-8) in the culture medium were then measured by ELISA. The results are the mean ± SD (n = 6), ** $P < 0.01$ vs. the control group, $^{\#\#}$ $P < 0.01$ vs. the PA group.

Supplementary Figure 2.

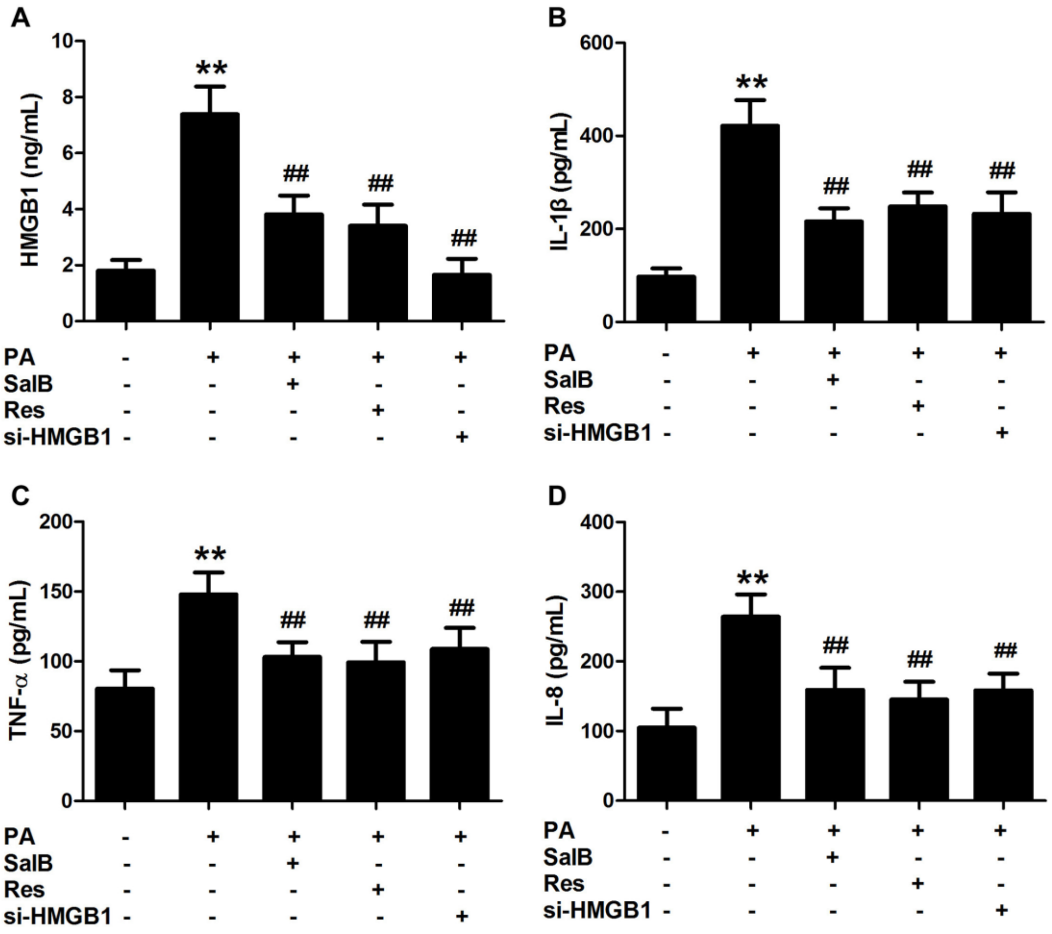

Supplementary figure 2. SalB, resveratrol, and HMGB1 siRNA decrease PA-induced hepatic inflammation. HepG2 cells were pretreated with 8 μM SalB, 10 μM resveratrol (Res), or HMGB1 siRNA and then exposed to PA for 24 h. The levels of (A) HMGB1, (B) interleukin-1β (IL-1β), (C) tumor necrosis factor-α (TNF-α), and (D) interleukin-8 (IL-8) in the culture medium were then measured by ELISA. The results are the mean ± SD (n = 6), ** *P* < 0.01 vs. the control group, $^{\#\#}$ *P* < 0.01 vs. the PA group.
